# Tin Mesoporphyrin Selectively Reduces Non-Small-Cell Lung Cancer Cell Line A549 Proliferation by Interfering with Heme Oxygenase and Glutathione Systems

**DOI:** 10.3390/biom11060917

**Published:** 2021-06-21

**Authors:** Valeria Sorrenti, Agata Grazia D’Amico, Ignazio Barbagallo, Valeria Consoli, Salvo Grosso, Luca Vanella

**Affiliations:** Department of Drug and Health Sciences, University of Catania, 95125 Catania, Italy; sorrenti@unict.it (V.S.); ignazio.barbagallo@unict.it (I.B.); valeria_consoli@yahoo.it (V.C.); salvogrosso@outlook.it (S.G.); lvanella@unict.it (L.V.)

**Keywords:** cancer, heme-oxygenase, porphyrins, oxidative stress

## Abstract

In order to maintain redox homeostasis, non-small-cell lung cancer (NSCLC) increases the activation of many antioxidant systems, including the heme-oxygenase (HO) system. The overexpression of HO-1 has been often associated with chemoresistance and tumor aggressiveness. Our results clearly showed an overexpression of the HO-1 protein in A549 NSCLC cell lines compared to that in non-cancerous cells. Thus, we hypothesized that “off-label” use of tin mesoporphyrin, a well-known HO activity inhibitor clinically used for neonatal hyperbilirubinemia, has potential use as an anti-cancer agent. The pharmacological inhibition of HO activity caused a reduction in cell proliferation and migration of A549. SnMP treatment caused an increase in oxidative stress, as demonstrated by the upregulation of reactive oxygen species (ROS) and the depletion of glutathione (GSH) content. To support these data, Western blot analysis was performed to analyze glucose-6-phosphate dehydrogenase (G6PD), TP53-induced glycolysis and the apoptosis regulator (TIGAR), and the glutamate cysteine ligase catalytic (GCLC) subunit, as they represent the main regulators of the pentose phosphate pathway (PPP) and glutathione synthesis, respectively. NCI-H292, a subtype of the NSCLC cell line, did not respond to SnMP treatment, possibly due to low basal levels of HO-1, suggesting a cellular-dependent antitumorigenic effect. Altogether, our results suggest HO activity inhibition may represent a potential target for selective chemotherapy in lung cancer subtypes.

## 1. Introduction

Lung cancer is the leading cause of cancer-related deaths worldwide [1]. During lung tumorigenesis, cancer cells enhance their metabolic output, which in turn increases the production of reactive oxygen species (ROS). Non-small-cell lung cancer (NSCLC) is the most common of its type, accounting for more than 80% of all lung cancers. To maintain oxidative homeostasis, non-small-cell lung cancer (NSCLC) increases the transcription of antioxidant genes [2]. The coordinated induction of antioxidant stress genes is regulated through a cis-acting element known as the antioxidant responsive element (ARE) within the regulatory regions of these genes [3]. Nrf2 (nuclear factor erythroid 2 (NFE2)-related factor 2) is a major regulator of ARE-mediated gene expression and belongs to the Cap-N-Collar family of transcription factors, forming heterodimers with small Maf proteins, subsequently binding to the ARE in target genes [4,5]. Heme oxygenase (HO) is regulated by the Nrf2/ARE pathway and is among the family of cellular antioxidant defense and detoxification enzymes, representing an important cytoprotective system to overcome oxidative stress and inflammation [6,7,8]. HO is a microsomal enzyme, which acts as a catalyst for the first rate-limiting step in the degradation of heme and plays an important role in the recycling of iron. It cleaves the *α*-meso carbon bridge of heme, yielding equimolar quantities of carbon monoxide, iron ions Fe^2+^, and biliverdin, which is subsequently converted into antioxidant bilirubin by biliverdin reductase [9,10].

The main isoforms of HO in mammals (HO-1 and HO-2) catalyze the same reaction, although they have different distribution and regulation [11,12]. It is known that HO-2 is constitutively expressed, whereas HO-1 is inducible by many structurally unrelated pharmacological and other agents, as well as by a variety of stimuli, such as inflammation or cellular stress [6,7,13,14]. 

The biological roles of HO-1 are believed to be associated with fundamental, adaptive, and innate defense responses against various cellular stress conditions, including oxidative stress. In addition to the anti-inflammatory and anti-apoptotic functions of HO-1, it is also known to play a role in the control of growth and proliferation in a cell-type-specific manner [11,15,16].

The use of synthetic heme analogues as competitive inhibitors of heme oxygenase activity represents a novel means of controlling severe hyperbilirubinemia in newborns. A series of preclinical and clinical studies demonstrated that the mesoporphyrin analogues are the most potent inhibitors, with tin mesoporphyrin (SnMP) proving to be the most efficacious with respect to safety and potency [17,18]. Nevertheless, very few published studies in the cancer field have used SnMP to target HO-1. The relevance of HO-1 in tumorigenesis has been demonstrated in several types of cancers, including some gastrointestinal cancers, glioma, melanoma, prostate cancer and hematological malignancies [19,20,21,22].

The increase in HO-1 expression prevents DNA damage as well as the initiation of carcinogenesis in normal cells. However, HO-1 overexpression promotes cancer cell proliferation and invasiveness in the late phase of tumorigenesis [23,24]. HO-1 protects cancer cells from apoptosis induced by chemotherapeutic agents or irradiation, suggesting its involvement in therapeutic resistance [25,26,27,28]. Many studies have shown that HO-1 can act as an immunomodulator, suppressing immune cell maturation, activation and infiltration. Additionally, HO-1 inhibits apoptosis through carbon monoxide production, leading to the suppression of the proapoptotic protein p53 [29].

Recent findings suggest that the tumor suppressor p53 plays a role in energy metabolism by regulating metabolic processes [30]. Furthermore, p53 activates TP53-induced glycolysis and the apoptosis regulator (TIGAR), which directs glucose to the pentose phosphate pathway (PPP) [31,32]. Glucose-6-phosphate dehydrogenase (G6PD) is the rate-limiting enzyme in the PPP, a metabolic pathway involving nucleic acid as a precursor and NAPDH synthesis. In addition, NAPDH is relevant to the maintenance of antioxidant defenses since it provides reducing equivalents for the maintenance of a pool of reduced glutathione to balance the redox state [33]. Upregulation of the G6PD level or activity is often observed in many kinds of cancer [34,35,36], suggesting that PPP may represent an important target for regulating the redox homeostasis in cancer.

The goal of the present study was to determine the effect of tin mesoporphyrin, a well-studied HO activity inhibitor, in reducing cell proliferation by affecting the PPP pathway and redox homeostasis.

## 2. Materials and Methods 

### 2.1. Cell Culture and Treatments

Experiments were conducted on human lung adenocarcinoma (A549) (ATCC CCL-185-LUC2), human mucoepidermoid carcinoma (NCI-H292) (ATCC CRL-1848) and human bronchial epithelial (BEAS-2B) (ATCC CRL-9609) cell lines purchased from the American Type Culture Collection (ATCC, Rockville, MD, USA). Cells were cultured in Dulbecco’s modified Eagle’s medium (DMEM), high glucose (HG) and RPMI 1640, supplemented with 10% FBS and 1% penicillin–streptomycin and maintained at 37 °C and 5% CO_2_. Cells were treated with 5 µM and 10 µM of tin mesoporphyrin (SnMP) and 10 µM of cobalt protoporphyrin IX (CoPP) for 72 h.

### 2.2. Western Blot Analysis

A preliminary analysis was performed to evaluate HO-1 basal expression in the BEAS-2B, A549 and NCI-H292 cell lines. Cells were harvested, and pellets were sonicated and centrifugated at 1500 rpm for 10 min at 4 °C to extract proteins from total lysate. Protein samples (80 µg) were diluted in 4× NuPage LDS sample buffer (Invitrogen, Waltham, MA, USA, NP0007), heated at 80 °C for 5 min and then separated by ExpressPlus™ PAGE Gel 12% acrylamide (GenScript, Piscataway, NJ, USA) with a Tris-MOPS running buffer (GenScript, Piscataway, NJ, USA) by electrophoresis. Proteins were then transferred to a PVDF membrane (Bio-Rad, Milan, Italy) using the TransBlot® SE Semi-Dry Transfer Cell (Bio-Rad, Milan, Italy), and blots were blocked using the Odyssey Blocking Buffer (LI-COR Biosciences, Lincoln, NE, USA) for 1 h at room temperature. Membranes were incubated overnight with HO-1 (GTX101147, diluted 1:1000, GeneTex, Irvine, CA, USA) and β-actin (GTX109639, diluted 1:7000, GeneTex) primary antibodies. Goat anti-rabbit secondary antibody was used to detect blots (dil. 1:7000). Blots were scanned, and densitometric analysis was performed with the Odyssey Infrared Imaging System (LI-COR, Milan, Italy). Values were normalized to β-actin. Western blot analysis was performed on A549 cells to evaluate the protein expression of HO-1, TIGAR, G6PD and GCLC after a 72 h treatment with 5 µM and 10 µM of SnMP. Primary antibodies against HO-1 (GTX101147, GeneTex), TIGAR (GTX110514, GeneTex), G6PD (sc-67165, Santa Cruz Biotechnology, Dallas, TX, USA) and GCLC (ab80841, Abcam, Cambridge, UK) were all used, diluted 1:1000. Subsequently, GCLC basal levels were evaluated in BEAS-2B and A549 cells. Secondary antibodies diluted 1:1000 were used, and blots were scanned as previously described [37]. Values were normalized to β-actin.

### 2.3. RNA Extraction and Quantitative Real-Time PCR Analysis

Upon reaching confluence, cells were harvested, and RNA extraction was performed using the Trizol reagent (Invitrogen, Carlsbad, CA, USA). First-strand cDNA was then synthesized with the Applied Biosystem (Foster City, CA, USA) reverse transcription reagent. Quantitative RT-PCR analysis was performed in Step One Fast Real-Time PCR System Applied Biosystems using the SYBR Green PCR Master Mix (Life Technologies, Monza MB, Italy) to evaluate the basal genes’ expression of HO-1 and then GCLC in both the A549 and BEAS-2B cell lines. Results were normalized with the housekeeping gene GAPDH using a comparative 2–ΔΔCt method. Primer sequences are listed in Table S1. 

### 2.4. Measurement of HO-1 Enzymatic Activity

BEAS-2B and A549 cells were harvested, and protein levels in the cell lysate were quantified to evaluate basal HO-1 enzymatic activity by measuring the bilirubin formation through the difference in absorbance at 464 to 530 nm. Reaction mixtures consisted of 20 mM Tris-HCl, pH 7.4, (2 mg/mL) cell lysate, 0.5–2 mg/mL biliverdin reductase, 1 mM NADPH, 2 mM glucose 6-phosphate (G6P), 1 U G6P dehydrogenase and 25 μM hemin. Incubation was carried out in a circulating water bath in the dark for 1 h at 37 °C. The reaction was stopped by adding chloroform. After recovering the chloroform phase, the amount of bilirubin that was formed was measured with a double-beam spectrophotometer at OD 464–530 nm (extinction coefficient, 40 mM/cm^−1^ for bilirubin). One unit of the enzyme was defined as the amount of enzyme catalyzing the formation of 1 nmol of bilirubin/mg protein/h. The same protocol was performed on A549 cells after a 72 h treatment with 5 μM and 10 μM of SnMP.

### 2.5. Viability Assay (MTT)

In order to test the effect of SnMP on cell viability, A549, BEAS-2B and NCI-H292 were seeded into 96-well plates at a density of 7.0 × 10^3^ cells/well in 100 µL of culture medium. After 24 h, cells were treated with SnMP (5 µM and 10 µM) and CoPP (10 µM) in a medium supplemented with 1% FBS for 72 h. Following treatment, 100 µL of 0.25 mg/mL 3-(4, 5-dimethylthiazol-2-yl)-2, 5-diphenyltetrazolium bromide (MTT) (ACROS Organics BV) and culture medium solution were added to each well, and cells were incubated for 2 h at 37 °C and 5% CO_2_. After incubation, the supernatant was removed, and 100 µL of DMSO was added to each well to dissolve formazan salts produced by mitochondria. The amount of formazan was proportionate to the number of viable cells in the sample. Finally, absorbance (OD) was measured in a microplate reader (Biotek Synergy-HT, Winooski, VT, USA) at λ = 570 nm. Eight replicate wells were used for each group.

### 2.6. Wound-Healing Assay

A549 cells were grown to confluence in six-well dishes (5 × 10^4^ cells/well) in 1 mL of complete medium. A scratch was made using a 200 μL pipette tip and wound closure followed; then, the cells were incubated in 1% serum medium with or without SnMP. Quantitative assessment of the wound area was performed under an inverted microscope, as previously described [38]. The closure of the scratch was viewed and imaged at 24 h, 48 h and 72 h. The migration was calculated as the average number of cells observed in three random, high-power wounded fields/per well in duplicate wells.

### 2.7. Thiol Group Determination

The concentration of non-protein thiol groups (RSH), reflecting about 90% of GSH cellular content, was measured by the total A549 cell lysate. This was obtained from abovementioned experimental conditions, using a spectrophotometric assay based on the reaction of thiol groups with 2,2-dithio-bis-nitrobenzoic acid (DTNB). DTNB solution was mixed with the sample and incubated for about 20 min in the dark until the noticeable appearance of a yellow color. After incubation, samples were centrifugated at 3000 rpm for 10 min at room temperature. The supernatant was collected and set in a black 96-well plate for measurement of absorbance in a microplate reader (Biotek Synergy-HT, Winooski, VT, USA) at λ = 412 nm. Results are expressed as pmoles/µL. Experiments were conducted in quadruplicate.

### 2.8. Measurement of ROS Levels

Levels of reactive oxygen species (ROS) were determined using fluorescent probe 2′, 7′-dichlorofluorescein diacetate (DCFH-DA). The cells were rinsed with a 0.1% Triton solution, which is required to enhance cellular probe permeation. Then, 100 µL of DCFH-DA working solution (200 µM) was added to each well and incubated at 37 °C for 30 min. After incubation, fluorescence was measured spectrofluorometrically (excitation, λ = 488 nm; emission, λ = 525 nm). ROS levels were measured in the presence and absence of 10 µM NADPH; specifically, it was added 3 h before the end of treatment. Results are expressed as fluorescence intensity (AU)/proteins (mg/mL). Eight replicate wells were used for each group.

### 2.9. Statistical Analysis

At least three independent experiments were performed for each analysis. The statistical significance (*p* < 0.05) of the differences between the experimental groups was determined by Fisher’s method for analysis of multiple comparisons. For comparison between treatment groups, the null hypothesis was tested by either a single-factor analysis of variance (ANOVA) for multiple groups or an unpaired t-test for two groups, and the data are presented as mean ± SEM.

## 3. Results and Discussion

Although many chemotherapeutics have been clinically employed to avoid the recurrence of malignancy after surgery, acquired chemotherapy resistance remains the biggest challenge to the successful treatment of patients with lung cancer [39,40].

Cancer cells often display elevated ROS as compared to their normal counterparts as a result of the accumulation of intrinsic and/or environmental factors. Several reports revealed that conditions inducing oxidative stress lead neoplastic cells to develop powerful antioxidant mechanisms, including the HO system [41]. In mammalian cells, HO-1 represents one of the most studied examples of a redox-regulated gene. Its transcriptional regulation is highly inducible by oxidative stress and involves several redox-sensitive transcriptional factors [42]. It is important to note that the metabolic status of cancer cells may affect HO-1 expression that is dependent on different signal pathways and transcription factors, suggesting a possible yet unclear regulation of HO-1 [43].

Several studies have highlighted the implications of HO-1 in carcinogenesis, since its overexpression affects and enhances tumor growth and proliferation in many human cancers, such as human lung adenocarcinoma [24,44,45]. The proliferative or anti-proliferative role of HO-1 in tumors seems to be highly tissue- and cell-specific. On one hand, HO-1 inhibition has been shown to reduce tumor growth in a mouse model of lung cancer and to inhibit proliferation in the A549 lung cancer cells [46,47]. Degese et al. showed HO-1 expression correlates with later stages of the disease and with lymph node metastasis in NSCLC [48]. Data reported by Ma et al. showed a higher resistance to non-thermal plasma exposure in A549 cells as compared to that in H1299 and H322 lung cancer cells. The obtained results may be linked to the highest basal level of HO-1 expression in A549 cells [49]. On the other hand, Tertil et al. identified a particular subtype of NSCLC where HO-1 acts as a tumor suppressor, inhibiting cancer cell proliferation, migration, tumor growth and angiogenesis [50,51].

Basal expression of HO-1 may be the key in the different mechanisms of drug response.

**Figure 1:**
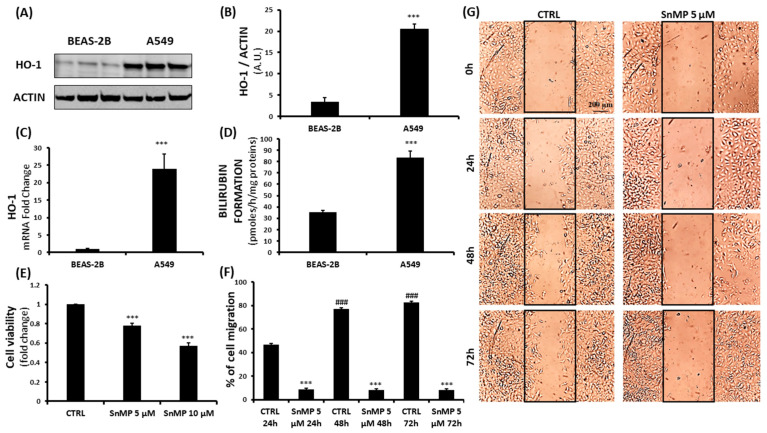
(**A**–**D**) Evaluation of HO-1 basal levels in healthy human bronchial epithelial (BEAS-2B) and human lung adenocarcinoma (A549) cell lines, investigating differences in protein content, gene expression and enzymatic activity (*** *p* < 0.001 vs. BEAS-2B). (**E**) Assessment of SnMP (5 µM, 10 µM) effect on A549 cell viability (*** *p* < 0.001 vs. CTRL). (**F**,**G**) SnMP effect on A549 cell migration rate. (^###^ *p* < 0.001 vs. CTRL 24 h, *** *p* < 0.001 vs. CTRL 24 h, 48 h and 72 h). Results are expressed as mean ± SEM.

Tin mesoporphyrin (SnMP) is a potent HO inhibitor that has undergone extensive clinical study for neonatal hyperbilirubinemia, without showing serious side effects [17]. Reducing the two vinyl groups to form ethyl groups, which at the C2 and C4 positions of the porphyrin macrocycle forms SnMP, greatly enhanced the potency of the metalloporphyrin, inhibiting HO activity and reducing bilirubin production. To investigate HO-1 levels, multiple assays (Figure 1) were conducted, leading to the observation of a significant HO-1 overexpression in the lung cancer cell line A549 when compared to levels in healthy human bronchial epithelial cells (BEAS-2B), as shown through Western blot and gene level analysis (Figure 1A–C). Moreover, HO enzymatic activity was evaluated by measuring bilirubin formation, as shown in Figure 1D, which further demonstrates hyperactivation of the HO system in these cancerous cells. According to these observations, we hypothesized the potential of an “off-label” use of tin mesoporphyrin, exploiting its inhibitory activity in an important antioxidant system for tumor cellular growth and invasiveness. MTT assay revealed a significant reduction in A549 cell viability after 72 h of SnMP treatment (Figure 1E). Specifically, doses of 5 µM and 10 µM led to a decrease of 22% and 43%, respectively. Exposing BEAS-2B to SnMP for 72 h caused a slight decrease in cell viability, as shown in Figure S1C, demonstrating that HO activity inhibition mainly affects cells that overexpress basal HO-1 levels.

**Figure 2:**
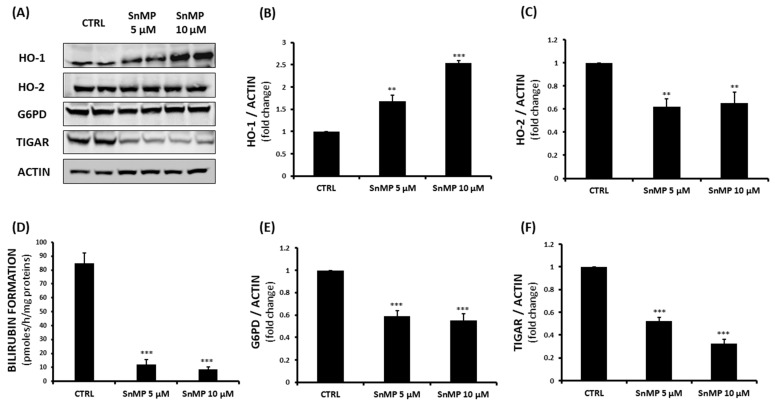
(**A**–**C**,**E**,**F**) Evaluation of SnMP (5 µM, 10 µM) effects on HO-1, HO-2, G6PD and TIGAR protein levels on A549 cells. (**D**) Measurement of HO enzymatic activity in A549 cells after 5 µM and 10 µM SnMP treatment (** *p* < 0.01, *** *p* < 0.001 vs. CTRL). Results are expressed as mean ± SEM.

Furthermore, to evaluate the effects of SnMP on cell migration, we performed a wound healing assay. As shown in Figure 1F,G, A549 cell motility was significantly increased after 48 h and 72 h in cells cultured with a complete medium with 1%FBS (^###^ *p* < 0.001 vs. CTRL 24 h), while the SnMP administration was able to counteract the cell migration rate by inducing a significant reduction in all experimental times (*** *p* < 0.001 vs. CTRL 24 h, 48 h and 72 h). Western blot analysis was performed to evaluate the effect of SnMP treatment on protein expression (Figure 2A); in particular, HO-1, HO-2, TIGAR and G6PD were analyzed. As shown in Figure 2B, inhibition of HO activity mediated by SnMP led to a significant increase in the HO-1 protein, as expected and previously shown by others [52].

According to Abate et al. [52], SnMP accelerates the degradation of Bach1, a negative transcriptional factor for heme oxygenase. The displacement of Bach1 leads to the recruitment of Nrf2 that binds to the HO-1 promoter by cooperating with the MafG and MafK proteins to activate HO-1 gene expression. Singh et al. clearly showed that high expression of NRF2-dependent antioxidant and metabolic genes is associated with reduced survival in patients with lung adenocarcinoma [53].

**Figure 3:**
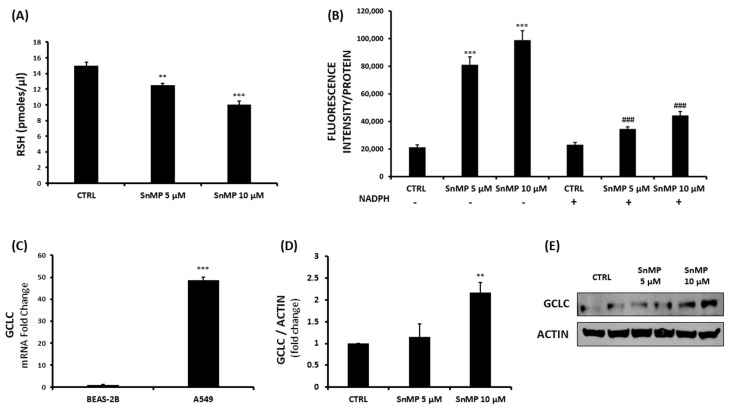
(**A**) Measurement of non-protein thiol group (RSH) concentration after SnMP (5 µM and 10 µM) treatment on A549 cells (** *p* < 0.01, *** *p* < 0.001 vs. CTRL). (**B**) Evaluation of the effect of SnMP on ROS levels with (+) or without (-) the addition of 10 µM NADPH in the A549 cell line (^###^ *p* < 0.001 vs. SnMP treatment, *** *p* < 0.001 vs. CTRL). (**C**) Determination of basal expression of the GCLC gene in A549 and BEAS-2B cell lines (*** *p* < 0.001 vs. BEAS-2B). (**D**,**E**) SnMP (5 µM, 10 µM) effect on GCLC protein levels on the A549 cell line (** *p* < 0.01 vs. CTRL). Results are expressed as mean ± SEM.

Our results show that HO-2 levels slightly decreased, suggesting a possible compensatory mechanism of the HO system following SnMP treatment (Figure 2C). Although HO-1 levels were increased following treatment, total HO activity was significantly reduced, as SnMP competes with heme as a substrate for heme oxygenase and inhibits enzymatic activity (Figure 2D). As cancer proliferation is often associated with an increased PPP, we chose to investigate the effect of SnMP on G6PD, the main protein involved in PPP and NADPH production, as well as TIGAR, a novel regulator of glucose metabolism. The results showed a decrease of up to 68% for the 10 µM SnMP treated group and more than a 40% reduction for both tested concentrations in G6PD and TIGAR levels (Figure 2 panels E and F respectively), demonstrating a significant effect of SnMP on PPP. The decreased NADPH content, as a result of the inhibition of PPP, is associated with a failure of restoring GSH. In order to evaluate GSH cellular content, thiol group determination via spectrophotometric assay was performed. This showed a gradual GSH reduction following SnMP treatment (Figure 3A), with a remarkable concurrent increase in ROS levels. However, as shown in Figure 3B, the addition of 10 µM NADPH reversed the effect of SnMP on ROS accumulation, indicating its role in redox system balance and NADPH production. mRNA and protein expression were assayed in both cell lines and basal conditions (Figure 3C and Figure S1A,B) in order to investigate the levels of the glutamate cysteine ligase catalytic subunit (GCLC), the first-rate limiting enzyme of glutathione synthesis. This highlighted the involvement of GSH in the delicate redox balance of the cancer cell microenvironment. As observed for the HO system, A549 cells showed a significant overexpression of GCLC compared to that of BEAS-2B cells, underlining the crucial role of multiple antioxidant systems in tumor cell survival. Subsequently, the effect of SnMP on GCLC protein levels was evaluated by Western blot analysis, showing a significant increase following 10 µM SnMP treatment (Figure 3D), which indicates a cellular response in attempt to deploy GSH content levels through its synthesis promotion.

## 4. Conclusions

The use of synthetic heme analogues, such as SnMP, to effectively inhibit the activity of heme oxygenase, the rate-limiting enzyme in bilirubin production, could represent an innovative approach to control cancer cell proliferation. This pharmacological approach may be cell specific. Referring to previous studies [51,54], we further investigated HO-1 expression in NSCLC cell lines A549 and NCI-H292 originating from adenocarcinoma and mucoepidermoid carcinoma, respectively. Western blot analysis revealed that A549 cells display a high basal expression of HO-1, while its levels are low in NCI-H292 cells (Figure S1D,E). Additionally, as shown in Figure S1F, SnMP treatment (10 µM) did not affect NCI-H292 cell viability. Contrary to SnMP, the addition of a well-known HO-1 inducer (CoPP) strongly decreased the cell viability of NCI-H292 cells. These conclusive results clearly demonstrate that each cell line responds differently to HO-1 modulation, suggesting that inhibition of HO activity may serve as a selective pharmacological tool in HO-dependent cells. The different antitumorigenic effect of SnMP in A549 and NCI H292 cells may involve gene level regulation by miRNA, as previously reported by others. According to Ciesla et al., HO-1 inhibition by SnPP reduced growth and vascularization of rhabdomyosarcoma in vivo, accompanied by the induction of miR-206 [55]. Additionally, stable HMOX1 overexpression, after plasmid transfection, inhibits tumorigenic and angiogenic capabilities of human NCI-H292 NSCLC cells through the downregulation of miR-378. Importantly, the pharmacological inhibition of HMOX1 with SnPPIX reversed the effects mediated by HMOX1 [51]. Altogether, our results suggest HO activity inhibition may represent a potential target for selective chemotherapy in lung cancer subtypes.

## Supplementary Materials

The following are available online at https://www.mdpi.com/article/10.3390/biom11060917/s1, Figure S1. (A,B) Evaluation of GCLC basal levels on BEAS-2B and A549 cell lines. (C) Assessment of SnMP effect on BEAS-2B cell viability. (D,E) HO-1 basal levels on NCI-H292 and A549 cell lines. (F) Effect of SnMP and CoPP on NCI-H292 cell viability. Table S1. Primers Sequence.
