# Supplementary material for: Tin Mesoporphyrin Selectively Reduces Non-Small-Cell Lung Cancer Cell Line A549 Proliferation by Interfering with Heme Oxygenase and Glutathione Systems
Source: Biomolecules. 2021 Jun 21;11(6):917. doi: 10.3390/biom11060917 (PMC8235249; doi:10.3390/biom11060917)
Supplement: Supplementary file 1 [file biomolecules-11-00917-s001.zip › biomolecules-1271079-supplementary.pdf]

Communication

SUPPLEMENTARY MATERIALS

Tin Mesoporphyrin selectively reduces the non-small-cell lung cancer cell line A549 proliferation by interfering with heme oxygenase and glutathione systems

Sorrenti V.[1], D'Amico A. G.[1,*], Barbagallo I.[1], Consoli V.[1], Grosso S.[1], Vanella L[1]

[1]Department of Drug and Health Sciences, University of Catania, Catania, Italy; e-mail@e-mail.com

*Correspondence: agata.damico@unict.it;

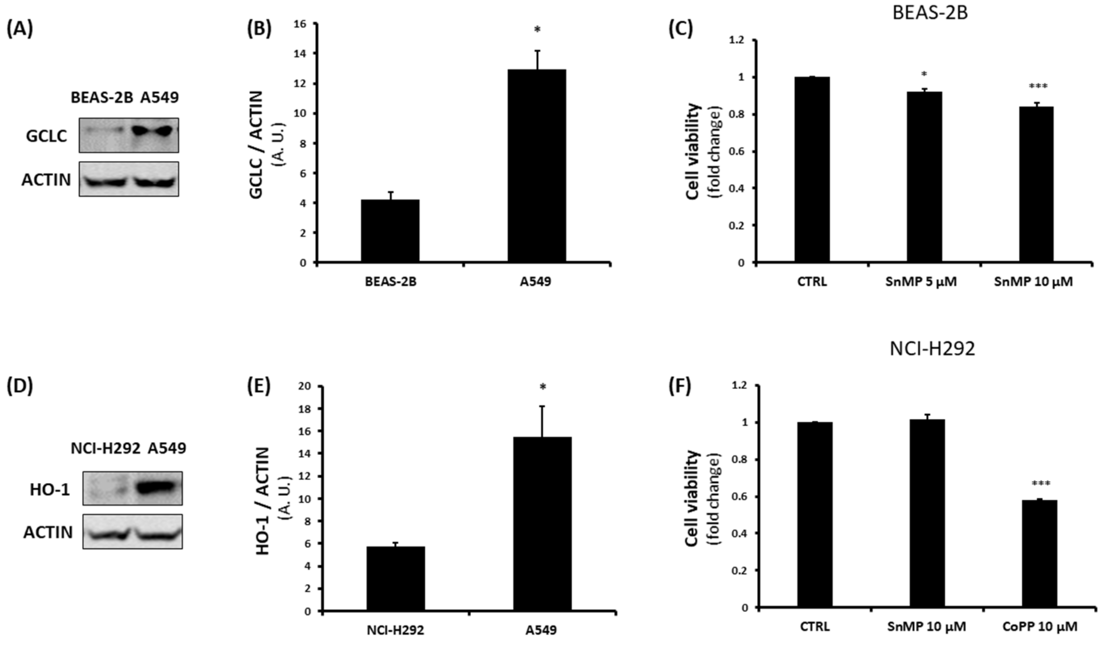

Figure S1. (**A,B**) Evaluation of GCLC basal levels on healthy human bronchial epithelial (BEAS-2B) and human lung adenocarcinoma (A549) cell lines (*$p<0.05$ vs BEAS-2B). (**C**) Assessment of SnMP (5 μM, 10 μM) effect on BEAS-2B cell viability (*** $p<0.001$, *$p<0.05$ vs CTRL). (**D,E**) HO-1 basal levels on human mucoepidermoid carcinoma (NCI-H292) and human lung adenocarcinoma (A549) cell lines (*$p<0.05$ vs NCI-H292). (**F**) Effect of SnMP (10 μM) and CoPP (10 μM) on NCI-H292 cell viability (*** $p<0.001$ vs CTRL). Results are expressed as mean ± SEM.

Table S1. Primers Sequence

Gene	Primer Forward	Primer Reverse
GAPDH	AGACACCATGGGGAAGGTGA	TGGAATTTGCCATGGGTGGA
GCLC	ACTTCATTTCCCAGTACCTTAACA	CCGGCTTAGAAGCCCTTGAA
HO-1	AAGACTGCGTTCCTGCTCAAC	AAAGCCCTACAGCAACTGTCG
